# Three-dimensional behavioural phenotyping of freely moving *C*. *elegans* using quantitative light field microscopy

**DOI:** 10.1371/journal.pone.0200108

**Published:** 2018-07-11

**Authors:** Michael Shaw, Haoyun Zhan, Muna Elmi, Vijay Pawar, Clara Essmann, Mandayam A. Srinivasan

**Affiliations:** 1 Department of Computer Science, University College London, London, United Kingdom; 2 Biometrology Group, National Physical Laboratory, Teddington, United Kingdom; 3 MIT TouchLab, Research Laboratory of Electronics and Department of Mechanical Engineering, Massachusetts Institute of Technology, Cambridge, Massachusetts, United States of America; Imperial College London, UNITED KINGDOM

## Abstract

Behavioural phenotyping of model organisms is widely used to investigate fundamental aspects of organism biology, from the functioning of the nervous system to the effects of genetic mutations, as well as for screening new drug compounds. However, our capacity to observe and quantify the full range and complexity of behavioural responses is limited by the inability of conventional microscopy techniques to capture volumetric image information at sufficient speed. In this article we describe how combining light field microscopy with computational depth estimation provides a new method for fast, quantitative assessment of 3D posture and movement of the model organism Caenorhabditis elegans (*C*. *elegans*). We apply this technique to compare the behaviour of cuticle collagen mutants, finding significant differences in 3D posture and locomotion. We demonstrate the ability of quantitative light field microscopy to provide new fundamental insights into *C*. *elegans* locomotion by analysing the 3D postural modes of a freely swimming worm. Finally, we consider relative merits of the method and its broader application for phenotypic imaging of other organisms and for other volumetric bioimaging applications.

## Introduction

Since it was proposed as a model organism several decades ago [1] the model organism *C*. *elegans* has become an important research tool used in many different fields from behavioural genomics [2] and neuroscience [3] to drug screening [4]. In particular, it is widely used in imaging-based behavioural studies in which the phenotypic effects of genetic mutations and external stimuli are assessed through variations in worm posture and locomotion [5]. Limitations in current imaging techniques mean that these experiments are typically restricted to two dimensional (2D) visualisation and analysis of worms crawling on the surface of an agar gel. However, even on a flat surface, to fully characterise aspects of behavioural response, such as head movement, requires visualisation and analysis of the shape and motion of the organism in three dimensions (3D). Further, the artificial setting of an agar plate fails to reproduce the complexity of the 3D environment inhabited by *C*. *elegans* in nature [6], limiting the freedom of the organism to express its full range of behaviours. Real time imaging of the organism in 3D offers the possibility of removing these constraints, allowing us to observe and quantify a greater range of behavioural phenotypes.

Despite significant developments in optical microscopy techniques in recent years [7], fast, minimally invasive volumetric imaging of biological systems remains challenging. Most conventional rapid 3D imaging techniques, such as light sheet microscopy [8] and spinning disk confocal microscopy [9] are inherently sequential, with an image volume built up from a series of image planes captured at different times. This fundamentally limits their ability to capture very fast dynamic processes, such as the unconstrained movement of *C*. *elegans*. Most of these methods also require the use of fluorescent labels to visualise the structure of interest. An alternative approach to fast 3D imaging is to capture multiple views of the sample simultaneously. In multifocal microscopy this is achieved using a curved diffraction grating which allows multiple focal planes to be imaged onto the camera simultaneously [10], however the number and separation of captured image planes is limited and correcting dispersion effects associated with broadband light sources can be challenging. Capturing images along two [11] or three [12] orthogonal view directions using multiple cameras has been shown to be effective for visualising the 3D posture and motion of *C*. *elegans*. However, such an approach requires a specialised optical set up with multiple synchronised cameras, along with associated alignment, calibration and image reconstruction processes. The need to illuminate and view the specimen clearly from multiple perpendicular view directions also imposes constraints on sample mounting and the experimental geometry.

Light field microscopy (LFM) [13] is an alternative volumetric imaging method, in which multiple perspective views of the sample are captured simultaneously through a single microscope objective lens. This is achieved using a microlens array (MLA) mounted at the native image (camera) plane, making it both simple and inexpensive to implement on a conventional widefield optical microscope system. Each raw camera exposure (light field image) contains both spatial and angular information, which can be manipulated to generate different perspective and focused views [14] of the object. LFM is suitable for viewing specimens under a range of imaging modalities including bright field, dark field and fluorescence [15]. In this article we describe how combining LFM with computational depth estimation methods, originally developed for photography [16], allows measurement of the 3D motion and body shape of *C*. *elegans* at a speed limited only by the frame rate of the camera. We apply this method to analyse 3D behavioural phenotypes by quantifying differences in posture and locomotion between two collagen mutants (*dpy-10* and *dpy-13*) freely moving within a 3D gel. By extending the eigenworm method of postural analysis [17] to 3D we use quantitative LFM to investigate the 3D swimming motion of a wild type worm. Finally, we discuss the broader application of quantitative LFM which, as a flexible imaging technique readily implemented on most standard research microscopes, offers new possibilities for experimental investigation of *C*. *elegans* and other dynamic 3D biological systems.

## Methods

### Light field microscope system

**Fig 1:**
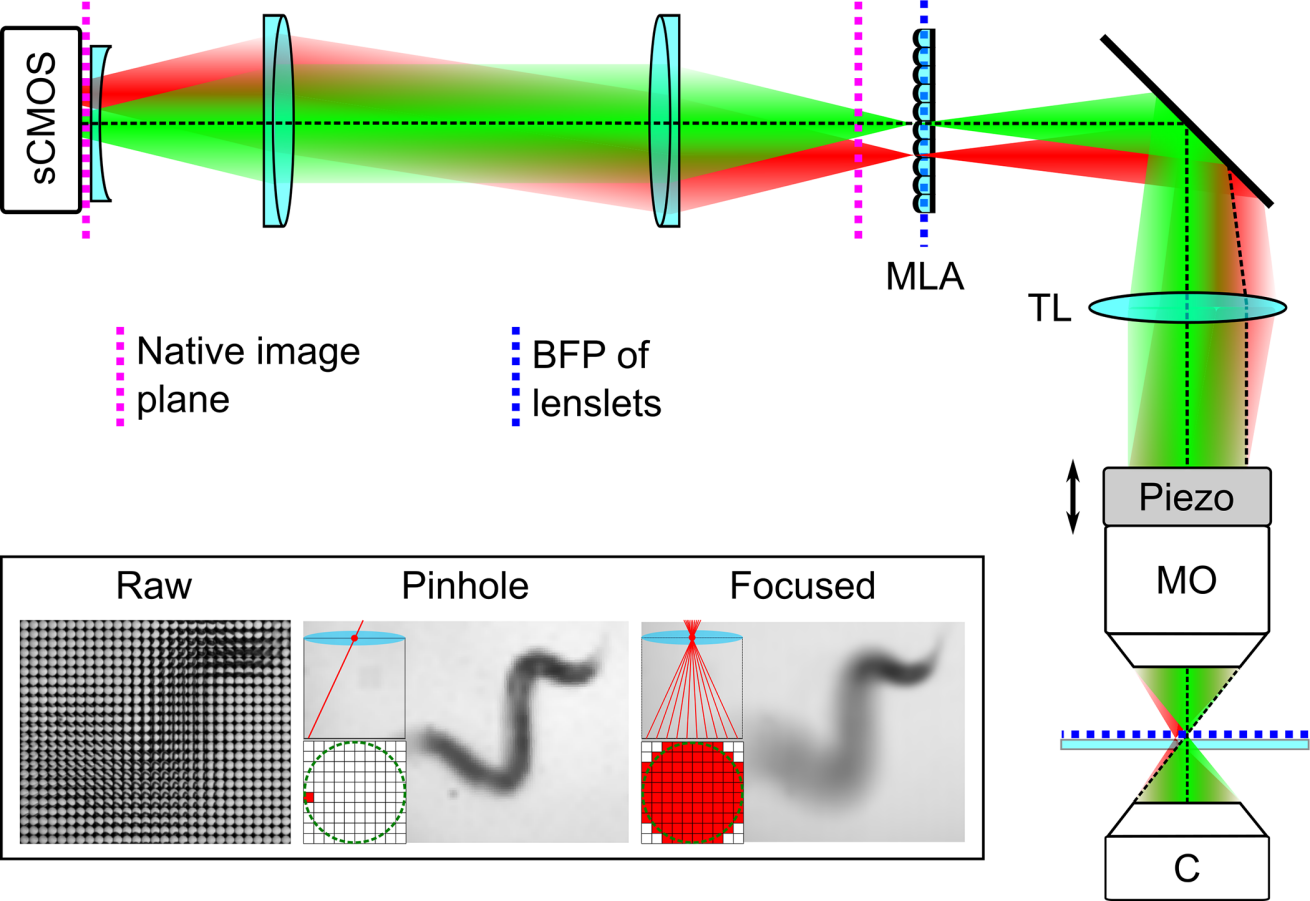
Light field microscope system used for 3D phenotypic imaging. Green and red ray bundles show how different lateral positions in the sample are focused onto different elements into the microlens array. Abbreviations: C–condenser lens; MO–microscope objective; piezo–piezoelectric nosepiece focusing stage; TL–tube lens; sCMOS–scientific CMOS camera. Inset shows (from left to right): A raw light field micrograph of a swimming C. elegans specimen; a perspective (pinhole) view of the object formed by extracting the same pixel from each microlens subimage; a focused view of the worm formed by summing the signal from all pixels within a microlens subimage. Pixels using to create each image are shown in red.

In order to capture 3D body shape and locomotion phenotypes of freely moving *C*. *elegans* we developed a custom LFM system by modifying a standard upright widefield microscope (BX51WI, Olympus) (Fig 1). The properties (lateral and angular resolution, depth of field and field of view) of an LFM system are strongly dependent on the parameters of the microlens array and its location in the imaging pathway (see supporting information S1 Text). For experimental and computational simplicity we adopt the ‘classic’ LFM configuration first described by Levoy et al. [13] by mounting an MLA (MLA-S125-F20, RPC Photonics) in the native image plane and projecting a 1:1 image of the back focal plane onto the image sensor of a scientific CMOS camera (ORCA-Flash 4.0v2, Hamamatsu Photonics) using a pair of achromatic doublet lenses (Thorlabs Inc.). Using a 10x/0.3 objective lens (UMPLFLN, Olympus) this gives a field of view of 1.3 mm x 1.3 mm. A field lens (a plano-concave singlet with a focal length of half the magnitude of the positive doublets) mounted immediately in front of the camera sensor was used to improve imaging of off axis image points by reducing Petzval field curvature. All these auxiliary components were mounted on an optical breadboard, supported on the same optical table as the microscope system. In this configuration each light field image (Fig 1—inset) is comprised of a tiled array of circular microlens subimages. Selecting the same pixel from each subimage creates a ‘pinhole’ view of the object from a corresponding view direction, whereas summing over all the pixels within each subimage results in an image focused at the native image plane. A captured light field *L*_*o*_ can be computationally refocussed, to simulate the effect of an axial shift in sample position, by shearing it parallel to the (*x*, *y*) plane using *L*_*α*_(*x*,*y*,*u*,*v*) = *L*_*o*_(*u*(1 − 1/*α*) + *x*/*α*,*v*(1 − 1/*α*) + *y*/*α*,*u*,*v*) [14]. This procedure, equivalent to laterally shifting each pinhole view prior to summation, allows computation of a focal series (z-stack) of images from a single camera exposure.

To increase image contrast and improve the reliability of image segmentation the microscope was configured for differential interference contrast imaging, with matched pairs of polarisers and Normarski prisms in the optical path before and after the sample. All images were captured at 100 frames per second (FPS), the maximum full frame rate of the camera, to minimise motion blur. For 3D reconstruction and behavioural analysis time lapse image sequences were down sampled to 20 FPS and exported as 16 bit multipage tiffs.

### Image reconstruction and depth estimation

All images were reconstructed and analysed using custom software written in MATLAB (MathWorks). Images were first corrected for vignetting, which decreases the detected radiance for off axis rays, by dividing each raw light field by a corresponding background image captured under identical illumination and detection conditions. The 2D light field image was then reshaped into a four-dimensional array, *L*_0_(*x*,*y*,*u*,*v*) using an automatic rectification procedure in which an initial estimate of the pitch and orientation of the subimages was from obtained by detecting the prominent peaks in a 2D Fourier transform of the raw image. This estimate was used to create a corresponding binary mask of tiled subimages. The mask parameters (pitch, orientation, lateral offset from centre), were then fine-tuned by translating and scaling the mask in to maximise the overlap (2D cross correlation) with the original normalised image light field image. The final mask settings were then used to resample the light field with an integer number of pixels in each subimage and reshape it into a 4D array.

Variation in image features with (post capture) changes in perspective and focus provide two ways of visualising the axial (*z*) position of objects within a light field image. The direction and magnitude of the apparent lateral shift in an object’s position with view direction (parallax) indicates how far it lies from the native object plane. Similarly, changes in sharpness of an image feature with computational refocusing indicate the position of the corresponding object relative to the native object plane. By analysing how these attributes of the light field change as it is computationally refocused (sheared parallel to the (x, y) plane) it is possible to determine the three-dimensional position of features within the sample. Importantly, the applied shear parameter, *α*, is related to the axial position in the sample by

$$\Delta z=P(1-1/\alpha )F/M^{2},$$ (1)

where *P* is the subimage pitch in camera pixels, *F* is the focal length of the MLA and *M* is the transverse magnification of the microscope.

In order to reconstruct the 3D body shape of the worm in each image frame we applied a depth estimation method originally developed for light field cameras [16], in which depth estimates based on defocus and correspondence are computed separately and then combined to produce a single (more robust) estimate. In practice this amounts to refocusing the light field and then computing defocus and correspondence responses as a function of the shear parameter *α* (see supporting information S1 Fig). A defocus depth response value is computed for each pixel in a refocused image, ${\bar{L}}_{\alpha }(x,y)=\sum_{u,v}L_{\alpha }(x,y,u,v)$ over a window *W*_*D*_ using, $D_{\alpha }(x,y)=1/|W_{D}|\sum_{(x^{\prime },y^{\prime })\in W_{D}}\left|{∆}_{xy}{\bar{L}}_{\alpha }(x^{\prime },y\prime )\right|$, where Δ_*xy*_ is the Laplacian operator. The correspondence depth response is defined as the average angular deviation in pixel value over a window *W*_*c*_, $C_{\alpha }(x,y)=\frac{1}{W_{c}}\sum_{(x^{\prime },y^{\prime })\in W_{c}}\sigma_{x^{\prime }y^{\prime }}$, where ${\sigma_{\alpha }}^{2}(x,y)=\frac{1}{N}\sum_{\left(u,v\right)}{\left(L_{\alpha }(x,y,u,v)-{\bar{L}}_{\alpha }(x,y)\right)}^{2}$. In both cases we found that a window size of five pixels gave optimal results. Defocus and correspondence depth estimates, *α*_D_^*^ and *α*_C_^*^ are defined as the maximum and minimum of the defocus and correspondence responses, ${\alpha_{D}}^{*}(x,y)=\arg\underset{\alpha }{\max}\;D_{\alpha }(x,y)$ and ${\alpha_{C}}^{*}(x,y)=\arg\underset{\alpha }{\min}\;C_{\alpha }(x,y)$. The final depth value is then computed by combining the depth estimates and corresponding confidence values, defined as the ratio of the defocus and correspondence response at the optimal depth to the corresponding value at the next best depth (next largest/smallest local maximum/minimum), using a Markov Random Fields optimisation [16].

**Fig 2:**
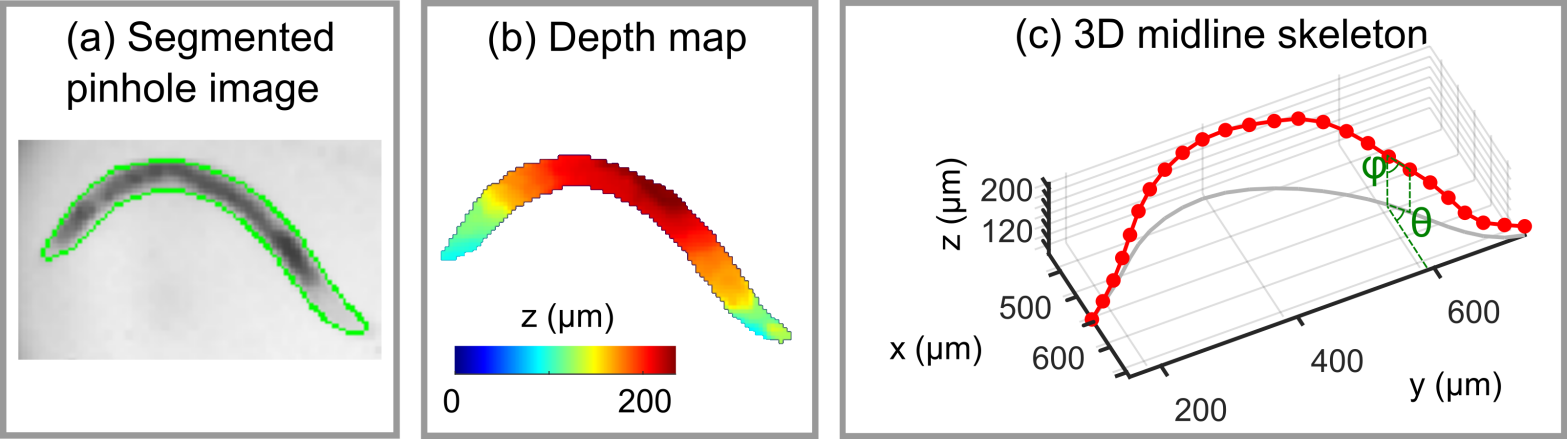
Extraction of *C*. *elegans* 3D midline skeleton from a light field image. (a) On axis pinhole view with segmentation mask outline shown in green. (b) Combined depth estimates within the segmentation mask displayed using a colour scale. (c) Final extracted midline skeleton of the organism comprised of 25 linear segments. The orientation of each segment is described by an azimuthal (**θ**) and polar angle (**ϕ**).

An estimate of the 3D body shape of the organism in a given image frame was computed by combining a depth image with a corresponding segmentation mask which defines the lateral footprint of the organism (projection along the z axis) (see Fig 2 and supporting information S1 Video). An approximate binary segmentation mask was generated from the on axis pinhole view by adaptive intensity thresholding, morphological hole filling and closing. This mask was then smoothed using active contours to create a final segmentation mask (Fig 2(A)). To improve the depth estimate a two frame temporal Kalman filter was applied to remove spurious depth values. A midline skeleton was then defined by fitting a smoothing spline to the 3D coordinates of pixels lying along a line from the nose to the tip of the tail through the centre of the mask (Fig 2(C)).

We found this method was effective for generating mid-line skeletons except when the worm adopted a self-occluded posture by coiling back across itself (see supporting information S2 Video). In self-occluded regions it was no longer possible to assign a single depth value to each lateral image pixel and both depth cues returned ambiguous results. For this reason, each time-lapse light field dataset was visually assessed prior to depth estimation and those containing self-occluded postures were excluded from further analysis.

By far the most computationally intensive part of the analysis was in computing the depth estimate for each image feature. The time taken for this step depended on a number of factors including the axial range over the depth responses are computed. On a desktop PC with an Intel Xeon E5-2690 v3 12 core 2.6 GHz processor and 64 GB of RAM, it took 24 seconds per time point to estimate depth of features over 1 mm, or approximately 8 minutes per second of video analysed at 20 frames per second. We anticipate that significant speed improvements should be possible by optimising software for computational efficiency. Further, the process is highly amenable to parallel processing and we anticipate that it could be speeded up considerably by exploiting the computing power of a graphics processor unit.

### Analysis of 3D posture and motion

To quantify posture and locomotion the metrics shown in Table 1 were computed directly from the 3D mid-line skeleton of the worm in each image frame. To compare behavioural phenotypes of *dpy* mutants these values were averaged over all time lapse image sequences.

**Table 1:** Posture and locomotion metrics computed to compare behaviour of dpy-10 and dpy-13 mutants.

**Centroid speed**	*CS*_*i*_ = |***r***_***i*+1**_ − ***r***_***i***_|/(*t*_*i*+1_ − *t*_*i*_), where ***r***_***i***_ is the centroid position vector at time *t*_*i*_.
**Non-planar deviation (NPD)**	*NPD* = *R*_3_/*R*_1_, where *R*_1_ and *R*_3_ are the lengths of the longest and shortest principal axes of the best fit ellipsoid to the worm posture, computed by performing principal component analysis on the corresponding mid-line skeleton.
**Curving rate for forward runs**	${CR}_{i}=\frac{1}{\left(t_{i+1}-t_{i}\right)}\cos^{-1}\left(\frac{\left|r_{i+1}∙r_{i}\right|}{\left|r_{i+1}||r_{i}\right|}\right),$ where ***r***_***i***_ is the centroid position vector at time *t*_*i*_.
**Directional autocorrelation for forward runs**	$D(n)=\frac{1}{N-n}\sum_{i=1}^{N-n}v_{i}∙v_{i+n}$, where v_*i*_ is the unit direction vector in the i^th^ frame and *N* is the total number of images in the sequence.

### Calculation of eigenworms and analysis of postural modes

Eigenworms were computed following the method described in [17]. Midline body skeletons were split into 25 segments, with the orientation of each segment described by a pair of angles (θ,ϕ) (Fig 2(C)). The mean of each of these angle vectors over the body length, 〈θ〉 and 〈ϕ〉, was set to zero, giving a shape representation which depends only on the posture adopted by the worm and rotation about its long (roll) axis. All 25 element postural angle vectors successfully extracted from midline skeletons for wild type, *dpy-10* and *dpy-13* worms moving in agarose and swimming wild type worms were concatenated, before eigenworms were computed using the MATLAB function ‘pca’. In practice, the close similarity between eigenworms computed from azimuthal and polar angle vectors allowed use of a single common set of eigenworms, which we chose as the azimuthal set. Eigenvalues for swimming motion were determined for each light field image frame by projecting the extracted midline skeleton onto this common eigenworm basis.

In contrast to alternative ways of describing 3D posture, such as the Frenet-Serret equations, we found this simple approach was relatively insensitive to noise in the midline skeleton and gave a robust and intuitive description of body shape. Computing a single set of eigenworms by combining azimuthal and polar angle vectors could in principle give a more efficient representation of the 3D posture, however the resulting projections are not simple to interpret and there remain questions about how best to normalise the angle vectors. As well as allowing a direct comparison with 2D postural mode analysis, treating the azimuthal and polar angles independently allows an independent validation of depth estimation by comparing corresponding azimuthal and polar eigenworms. Computing separate lateral and axial postural modes may also be important for particular behavioural assays [18].

### *C*. *elegans* culture and preparation

*C*.*elegans* strains, N2 (wild-type), CB128 (*dpy*-10) and CB184 (*dpy*-13), were obtained from the Caenorhabditis Genetics Center (University of Minnesota) and maintained according to standard procedure [1]. For experimental purposes, L4 staged animals were transferred to fresh plates a day prior to imaging (by which time they had reached adult stage). To visualise organisms moving in 3D, worms were prepared in an agarose gel (at 0.25% wt/vol in M9 buffer solution [19]) on a microscope coverslip.

To calibrate the depth scaling factor a single worm was paralysed by treatment with BDM (2,3-butanedione monoxime), diluted to 15 mg/ml in M9 buffer, for 60 minutes. For visualising swimming behaviour several drops of M9 buffer solution were added to an agarose pad containing a number of wild-type adults, causing worms to detach from the surface. A time lapse light field sequence of duration 30 seconds was captured beginning as soon as an animal was positioned close to the centre of the field of view of the microscope.

## Results

### Calibration of depth scaling factor

**Fig 3:**
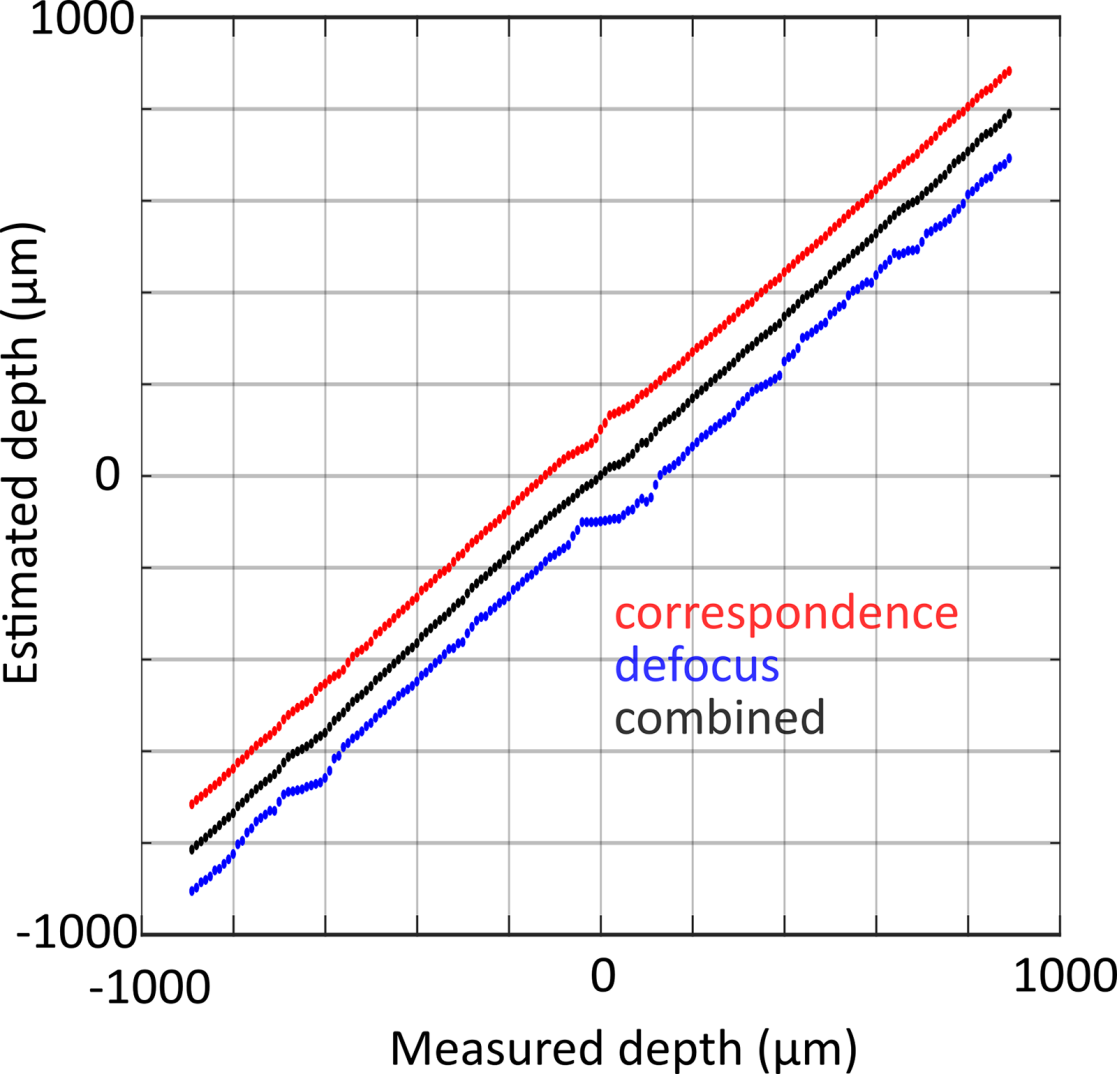
Calibration of depth scaling factor. Correspondence (red), defocus (blue) and combined (black) depth estimates versus known depth obtained by capturing a series of light field images as a paralysed *C*. *elegans* specimen was translated through a series of known axial positions. Defocus and correspondence estimates offset by + 100 μm and -100 μm for clarity.

The depth estimation accuracy was tested using a series of light field images of a paralysed *C*. *elegans* specimen mounted on a microscope coverslip captured as the microscope focusing stage was displaced by known amounts. Fig 3 shows the estimated depth (averaged over the midline of the worm) versus known focus offset (measured depth), where defocus and correspondence estimates have been offset by +/- 100 μm for clarity. Both correspondence, defocus and combined depth estimates vary linearly with real depth (R^2^ ≥ 0.9989 for all three) over the entire measured range of +/- 1 mm. The gradients of individual depth estimates are less than one, which we attribute to differences between the nominal and real system parameters used to relate the light field shearing parameter (*α*) to depth. All subsequent final depth estimates were scaled by the gradient of the fit to the combined depth estimate (0.883). The root mean square error of the linear fit to the combined estimate is 11.1 μm, which is a measure of the depth sensitivity (axial resolution) of the system. This value is considerably smaller than both the DOF of the refocused images (65 μm) and the total thickness of the worm body (approximately 50–70 μm for an adult), thus the depth value assigned to a given part of the worm corresponds to a contrast weighted average over the total worm thickness.

### Real time volumetric imaging of *C*. *elegans*

**Fig 4:**
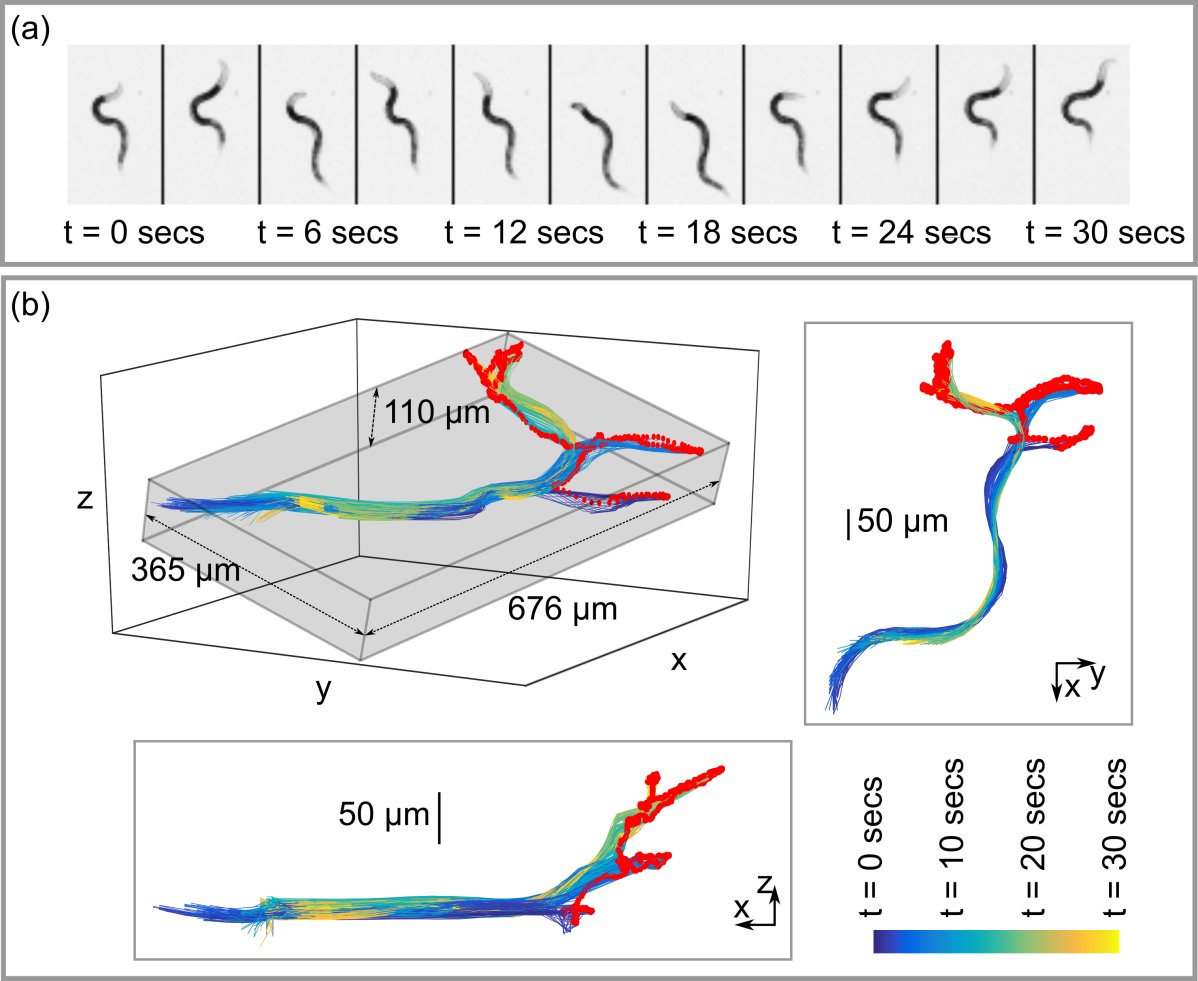
3D posture and movement of *C*. *elegans* during foraging. (a) On axis pinhole views from a 30 second long light field image sequence of a wild type *C*. *elegans* organism moving within agarose gel. (b) Perspective views showing corresponding reconstructed mid-line skeletons. The total volume occupied by the animal during the sequence, represented by the grey bounding box, is 0.027 mm^3^. The nose (red) explores a volume of 0.009 mm^3^.

For an initial proof of principle test, we captured a 30 second long time lapse light field image sequence of an adult wild type worm moving inside a block of agarose gel. During this period the worm underwent four phases of forward locomotion to reach different locations within the gel. At the end of each forward phase the worm paused briefly and executed a series of exploratory head movements (foraging) before reversing. Fig 4(A) shows on axis pinhole views of the worm in three second intervals. Fig 4(B) shows all 600 mid-line skeletons extracted from the light field images captured during this period (nose tip indicated in red). The grey bounding box shown in the isometric projection in the upper left part of Fig 4(B) indicates the total volume of 3D space visited by the worm during the sequence (0.027 mm^3^). The xy and xz projection views clearly illustrate the 3D movement of the head during the foraging phase, with the nose exploring a volume of 0.009 mm^3^ during the sequence. Such 3D information is a useful addition to existing methods for detecting and analysing foraging behaviour [20].

### Quantitative comparison of 3D behavioural phenotypes of cuticle mutants

**Fig 5:**
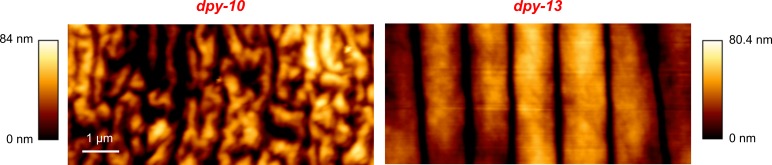
The cuticle topography of *dpy-10* and *dpy-13* captured using atomic force microscopy. Topography images of immobilised young adults captured in contact mode using a 2 nm probe tip as described in [21].

As a proof of principle experiment we applied quantitative light field microscopy to analyse the behaviour of two *C*. *elegans* cuticle mutants, *dpy-10* and *dpy-13*. We have previously studied these two mutants and found significant differences in cuticle topography [21]. Both have mutations in genes encoding collagen proteins, similar gross morphologies and are shorter and fatter than wild-type worms. However, whilst the cuticle of *dpy-13* contains regular annuli and furrows, *dpy-10* lacks this organised structure (see Fig 5). The cuticle of *C*. *elegans* has an important role in the animal’s ability to move in complex environments such as soil [22, 23]. The functional role of the annuli are not known, but are thought to permit flexibility during movement [22, 24]. In order to elucidate whether the absence of the annuli structure alters posture and locomotion in 3D environment, we compared dpy-10 and dpy-13 worms moving within agarose gel.

For each data set imaging was begun immediately after the application of a gentle mechanical stimulus (tap) to the sample mount to stimulate movement of the worm. Time lapse light field images were then acquired until the worm moved outside the field of view of the microscope. In total we recorded and analysed 27 separate time lapse image sequences of seven different *dpy-10* animals with a total duration of 9.2 minutes and 19 image sequences of 10 different *dpy-13* animals with a total duration of 4.4 minutes.

**Fig 6:**
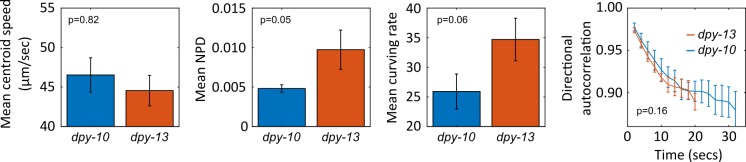
Mean locomotion and posture metrics for *dpy-10* (blue) and *dpy-13* (orange) mutants moving in an agarose gel. Error bars show ± standard error in the mean. Statistical significance of difference between measured values for each strain (confidence in rejection of the null hypothesis) indicated by the *p*-value shown in each plot. *P*-values computed using a two sample t-test (assuming unequal variances) applied to values measured for each strain, with the measured value for each individual worm was computed as the mean over all data captured for that animal. Statistical significance in difference between directional autocorrelation curves assessed by performing a two sample t-test on the decay constants of exponential fits applied to the mean data for each individual worm.

To quantify the posture and locomotion of the worms in each image sequence we computed the centroid speed, non-planar deviation (NPD) and curving rate in each image frame and the directional autocorrelation during forward runs (for details see Table 1). NPD is a measure of how far the worm’s posture extends away from a best fit plane and quantifies the ability of the worm to bend simultaneously in two perpendicular directions, whilst curving rate and directional autocorrelation describe how rapidly the worm changes its direction of motion. The averaged results (Fig 6) indicate that both mutants move with a similar speed, with mean centroid speeds of 46.1 ± 5.7 μm/sec and 47.0 ± 6.7 μm/sec for *dpy-10* and *dpy-13* respectively. Both the mean curving rate and NPD are substantially lower for *dpy-10*, suggesting that the lack of regular annuli and furrows in the cuticle limit the worm’s ability to rapidly change direction and adopt complex postures and supporting the theory that the cuticle annuli give flexibility to the worm during movement. This is also suggested by the more rapid decay in the directional autocorrelation curve for *dpy-13* and exponential fits (*D*(*t*) = *Ae*^−*bt*^ to the data yield decay constants (b) of 0.20 ± 0.04 and 0.33 ± 0.08, however these differences are not statistically significant (p = 0.16).

### 3D postural modes of swimming animals

**Fig 7:**
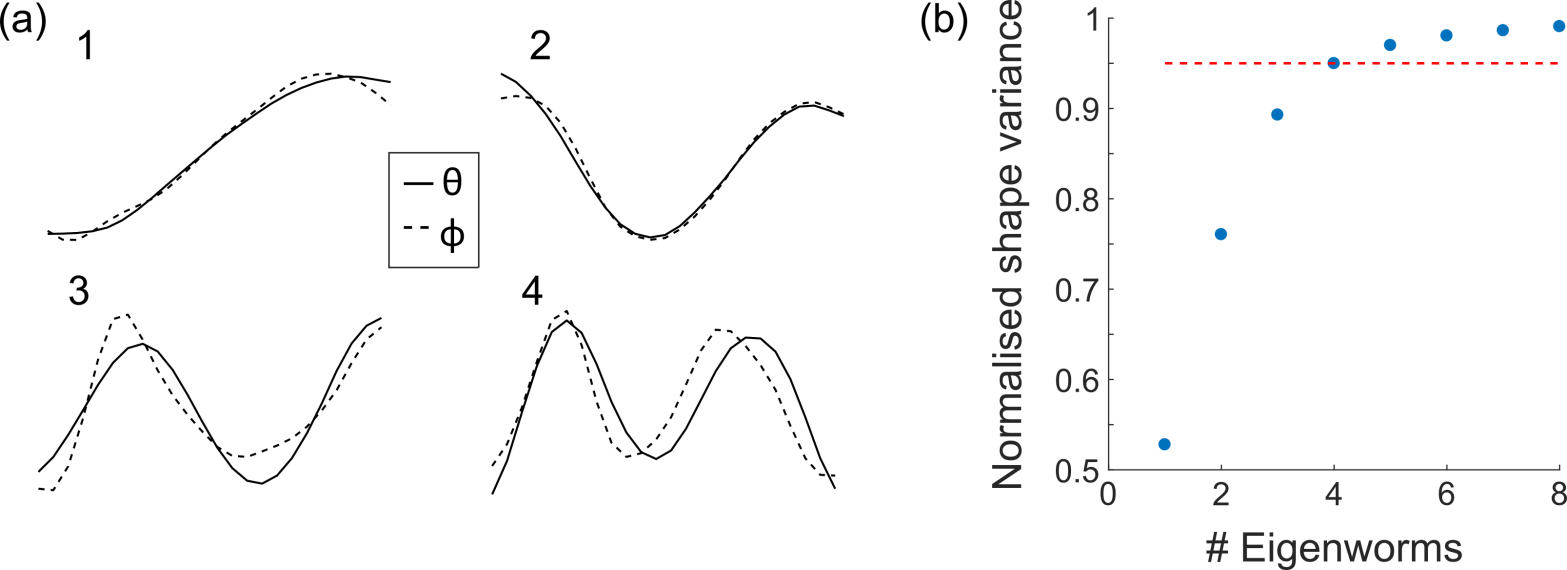
3D eigenworms. (a) The first four eigenworms computed separately from azimuthal (θ, solid line) and polar (ϕ, dashed line) angles. (b) Normalised shape variance using different numbers of eigenworms. The first four eigenworms in both θ and ϕ are sufficient to capture 95% of the body shape variance.

An alternative method to analyse worm behaviours is by describing the associated body shapes in a space of fundamental postures [2]. In this case, a 2D worm posture is represented by a vector of tangent angles describing the orientation of different sections of the mid-line skeleton. Performing principal component analysis on a large set of these tangent vectors, derived from time-lapse images, yields a set of fundamental postural modes or eigenworms [17]. Projecting a given midline skeleton onto this basis allows the posture to be described in terms of the associated projection amplitudes or eigenvalues. By capturing the 3D worm bodyshape quantitative light field microscopy allows this method to be extended to measure the 3D postural modes of freely moving worms. We achieved this by representing each reconstructed 3D midline skeleton as a pair of 25 element vectors *θ*(*s*) and *ϕ*(*s*) containing azimuthal and polar tangent angles. We generated 3D eigenworms from 3D midline skeletons for *dpy-10*, *dpy-13* and wild-type worms in agarose. Fig 7(A) shows the first four eigenworms derived from azimuthal and polar tangent angle vectors. Note the close resemblance between azimuthal and polar eigenworms which may be considered a further validation of the accuracy of the depth estimation method. This close similarity also means that in practice a single set of eigenworms (derived from either the azimuthal or polar tangent vectors) can be used to represent motion in lateral and axial directions. Similarly to previous work based on 2D eigenworms [17], our results indicate that 95% of the body shape variation is captured using the first four eigenworms in *θ* and *ϕ* (Fig 7(B)).

**Fig 8:**
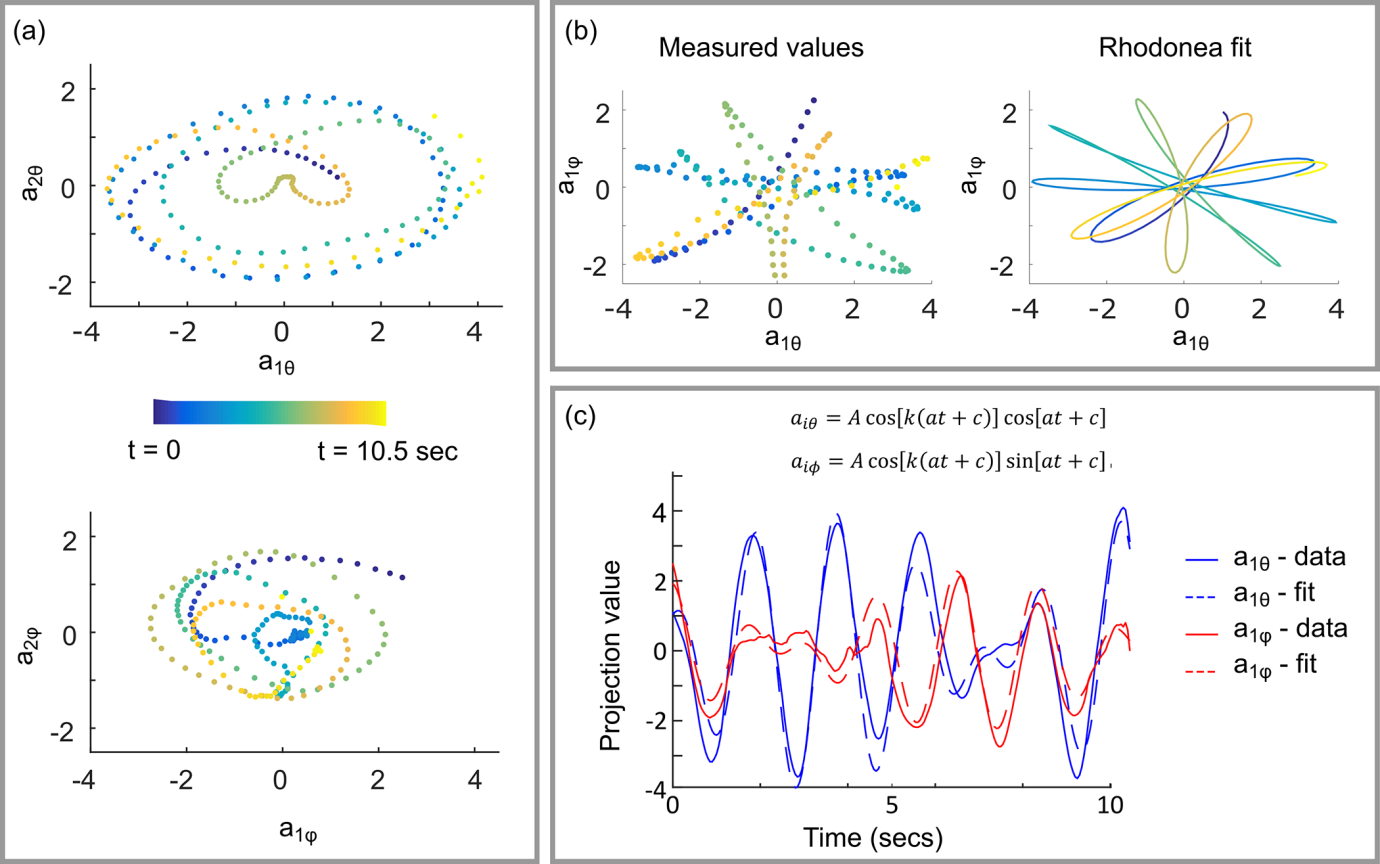
Time dependence of eigenworm projection amplitudes for a swimming wild-type worm. (a) Relationship between the first and second eigenvalues computed for azimuthal, θ (top) and polar, φ (bottom) angles. (b) Variation in projection amplitudes in azimuthal and polar angles for the first eigenworm (left) can be approximated using a Rhodonea curve (right). (c) A least squares fit to the Rhodonea indicates that the frequency with which the animal reorients its plane of oscillation is approximately 8.5 times lower than the periodic in plane motion of the animal during swimming.

Analysis of 2D eigenworms [17] has shown that the joint probably density function of the first two eigenworms forms a ring structure, indicating that the first two eigenworms form an oscillator with an approximately fixed amplitude and varying phase describing the basic crawling behaviour of the animal. We observe a similar result in 3D, however allowing the animal to move in 3D means that amplitude of the oscillator varies with time (depending on the orientation of the animal) and the resulting trajectory in the (*a*_1_,*a*_2_) plane is elliptical. Fig 8(A) shows examples of these elliptical trajectories for a wild type *C*. *elegans* swimming in buffer solution. We can gain further insight into the 3D swimming motion of this animal by considering the relationship between the corresponding θ and φ projection amplitudes. The top left panel in Fig 8(B) shows the first θ and φ eigenvalues for the same swimming wild type animal. The data bear a striking resemblance to the rose or rhodonea curve, *r* = *A* cos(*kt* + *c*), suggesting that we can write,

aiϕ=Acos[k(at+c)]cos[at+c]aiϕ=Acos[k(at+c)]sin[at+c]. (2)

Performing least squares fits to Eq 2 yields a good match to experimental data (Fig 7(C) and 7(D)), indicating that this simple expression effectively describes the 3D swimming motion of the organism. If the first cosine term in each expression is associated with the ventral-dorsal oscillatory motion of the worm body during swimming, then the second term describes the frequency with which the animal changes the orientation of this plane of oscillation. Independent fitting to the experimental data for the first and second eigenworms yields a mean value for *k* of 8.5 with a standard deviation of 0.3. To our knowledge this is the first time that this ‘rolling frequency’ has been experimentally measured.

## Discussion

We have shown that combining LFM with computational depth estimation allows quantification of 3D postural and locomotion characteristics in *C*. *elegans*, permitting extended phenotypic imaging of the organism in a more natural setting. In contrast to other 3D microscopy techniques, light field microscopy is readily implemented on many of the widefield microscope systems currently in use using widely available and relatively inexpensive optical components. The relative simplicity and flexibility of the technique make it attractive for quantitative 3D imaging of a range of other dynamic biological systems including other model organisms, such as the *Drosophila* embryo, and 3D cell cultures and organoids. The capability of quantitative LFM to capture 3D sample morphology from label free images makes it particularly attractive for long duration timelapse imaging of sensitive biological samples for which the use of fluorescent labels and high intensity laser excitation induces undesirable phototoxic effects [25]. However, LFM is also suitable fast volumetric fluorescence imaging and has been used for neural activity imaging in *C*. *elegans* and other organisms using fluorescent calcium indicators [26, 27]. This potential to capture 3D behavioural and neural activity information simultaneously opens up exciting new possibilities in experimental neurobiology. The primary limitation of LFM is its relatively modest spatial resolution, which stems from the need to sacrifice lateral spatial information in order to simultaneously capture multiple perspective views, and this has limited the application of the technique. There are, however a number of strategies for increasing spatial resolution. Firstly, changing the properties of the microlens array allows a trade-off between the spatial and angular sampling rates and the depth over which post capture refocusing is possible. Actively maintaining the object within the field of view using a closed loop object tracking system [28] would allow longer duration time-lapse imaging, permitting a reduced field of view and the use of higher magnification objective lenses for improved spatial sampling and achievable resolution. Exploiting the higher spatial sampling rate away from the native image plane by reconstructing light field images using 3D deconvolution [29], offers another way to improve spatial resolution. However, in preliminary experiments using this latter approach we found that the strong dependence of the spatial resolution on object depth and the presence of deconvolution artefacts caused errors in depth estimation. With further development of the depth estimation algorithms it may be possible to overcome these limitations. Recent work [30, 31] has shown mounting the microlens array in the pupil plane of the microscope objective rather than the image plane can improve lateral spatial resolution. Given the limited number of camera pixels this typically comes at the expense of fewer angular samples, but it would be an interesting exercise to compare the ability of these different LFM configurations to capture and reconstruct 3D samples structures.

A further technical limitation of LFM stems from the relatively limited angular baseline, which makes depth estimation and 3D reconstruction of complex, self-occluded postures, such as coils and knots, difficult. Recent work [32] has shown that matching a 2D image to a superposition of eigenworm postures offers a way to track coiled body shapes and such an approach may prove useful for reconstructing some self-occluded 3D body shapes.

## Supporting information

S1 Text Spatial resolution and working volume in LFM. A discussion of how microscope design parameters affect the lateral spatial resolution, field of view and effective depth of field in a light field microscope.
(DOCX)

S1 Fig Schematic illustration of computational depth estimation process. The central part of the figure depicts the processing of light field shearing in 3D (equivalent to laterally shifting each pinhole view). The left panel shows the refocused images created by integrating the sheared light field over all view directions (u, v). The right panel shows three 2D epipolar images, with each row of pixels corresponding to a line profile through a single pinhole image. When the light field is sheared by the amount corresponding to the depth of the object, its position does not change with view angle and it appears as a vertical line in the epipolar image. In this example defocus and correspondence responses (blue and red curves), both indicate the worm body is offset by ~-200 μm from the native object plane.
(TIF)

S1 Video Lateral segmentation and depth map for a freely swimming wild-type worm. Left: on axis pinhole view (grayscale) and segmented outline (yellow) of a wild-type worm swimming in buffer solution. Right: corresponding combined depth map. Images displayed at 20 frames per second.
(MP4)

S2 Video Examples of self-occluded postures adopted by *C*. *elegans*. Each image sequence shows the on axis pinhole views computed from part of a longer duration light field time lapse image set of a wild-type worm moving in agarose gel. In self-occluded (overlapping) regions it is no longer possible to assign a single depth value to each lateral image pixel and both depth cues return ambiguous results preventing reliable 3D reconstruction.
(MP4)

## References

[1] Brenner S. The Genetics of CAENORHABDITIS ELEGANS. Genetics. 1974;77(1):71–94. PMC1213120. 4366476 10.1093/genetics/77.1.71 PMC1213120

[2] Brown AEX, Yemini EI, Grundy LJ, Jucikas T, Schafer WR. A dictionary of behavioral motifs reveals clusters of genes affecting Caenorhabditis elegans locomotion. Proceedings of the National Academy of Sciences of the United States of America. 2013;110(2):791–6. doi: 10.1073/pnas.1211447110. PMC3545781. 23267063 10.1073/pnas.1211447110 PMC3545781

[3] Sengupta P, Samuel ADT. C. elegans: a model system for systems neuroscience. Current opinion in neurobiology. 2009;19(6):637–43. doi: 10.1016/j.conb.2009.09.009. PMC2904967. 19896359 10.1016/j.conb.2009.09.009 PMC2904967

[4] O'Reilly LP, Luke CJ, Perlmutter DH, Silverman GA, Pak SC. C. elegans in high-throughput drug discovery. Advanced Drug Delivery Reviews. 2014;69–70:247–53. https://doi.org/10.1016/j.addr.2013.12.001. doi: 10.1016/j.addr.2013.12.001 24333896 10.1016/j.addr.2013.12.001 PMC4019719

[5] Yemini E, Jucikas T, Grundy LJ, Brown AEX, Schafer WR. A database of C. elegans behavioral phenotypes. Nature methods. 2013;10(9):877–9. doi: 10.1038/nmeth.2560. PMC3962822. 23852451 10.1038/nmeth.2560 PMC3962822

[6] Frézal L, Félix M-A. C. elegans outside the Petri dish. eLife. 2015;4:e05849 doi: 10.7554/eLife.05849 25822066 10.7554/eLife.05849 PMC4373675

[7] Sydor AM, Czymmek KJ, Puchner EM, Mennella V. Super-Resolution Microscopy: From Single Molecules to Supramolecular Assemblies. Trends in Cell Biology. 2015;25(12):730–48. doi: 10.1016/j.tcb.2015.10.004 26546293 10.1016/j.tcb.2015.10.004

[8] Keller PJ, Schmidt AD, Wittbrodt J, Stelzer EHK. Reconstruction of Zebrafish Early Embryonic Development by Scanned Light Sheet Microscopy. Science. 2008;322(5904):1065–9. doi: 10.1126/science.1162493 18845710 10.1126/science.1162493

[9] Gräf R, Rietdorf J, Zimmermann T. Live Cell Spinning Disk Microscopy In: Rietdorf J, editor. Microscopy Techniques. Berlin, Heidelberg: Springer Berlin Heidelberg; 2005 p. 57–75. 10.1007/b102210 16080265

[10] Abrahamsson S, Chen J, Hajj B, Stallinga S, Katsov AY, Wisniewski J, et al Fast multicolor 3D imaging using aberration-corrected multifocus microscopy. Nat Meth. 2013;10(1):60–3. doi: 10.1038/nmeth.2277 23223154 10.1038/nmeth.2277 PMC4161287

[11] Kwon N, Pyo J, Lee S-J, Je JH. 3-D Worm Tracker for Freely Moving C. elegans. PLOS ONE. 2013;8(2):e57484 doi: 10.1371/journal.pone.0057484 23437394 10.1371/journal.pone.0057484 PMC3578814

[12] Kwon N, Hwang AB, You Y-J, V. Lee S-J, Ho Je J. Dissection of C. elegans behavioral genetics in 3-D environments. Scientific Reports. 2015;5:9564 doi: 10.1038/srep09564. PMC4424945. 25955271 10.1038/srep09564 PMC4424945

[13] Levoy M, Ng R, Adams A, Footer M, Horowitz M. Light field microscopy. Acm Transactions on Graphics. 2006;25(3):924–34. doi: 10.1145/1141911.1141976. WOS:000239817400054.

[14] Ng R. Fourier slice photography. Acm Transactions on Graphics. 2005;24(3):735–44. doi: 10.1145/1073204.1073256. WOS:000231223700044.

[15] Levoy M, Zhang Z, McDowall I. Recording and controlling the 4D light field in a microscope using microlens arrays. Journal of Microscopy. 2009;235(2):144–62. WOS:000268300100005. doi: 10.1111/j.1365-2818.2009.03195.x 19659909 10.1111/j.1365-2818.2009.03195.x

[16] Tao MW, Hadap S, Malik J, Ramamoorthi R. Depth from Combining Defocus and Correspondence Using Light-Field Cameras. Proceedings of the 2013 IEEE International Conference on Computer Vision. 2013:673–80. doi: 10.1109/iccv.2013.89

[17] Stephens GJ, Johnson-Kerner B, Bialek W, Ryu WS. Dimensionality and Dynamics in the Behavior of C. elegans. PLOS Computational Biology. 2008;4(4):e1000028 doi: 10.1371/journal.pcbi.1000028 18389066 10.1371/journal.pcbi.1000028 PMC2276863

[18] Magnes J, Raley-Susman KM, Jago A, Spuhler K, Wine D, Kpulun T. Gravity Studies of C. elegans. Biophysical Journal. 2013;104(2, Supplement 1):669a https://doi.org/10.1016/j.bpj.2012.11.3694.

[19] Stiernagle T. Maintenance of C. elegans. In: Community TCeR, editor. WormBook: WormBook; 2006. 10.1895/wormbook.1.101.1 PMC4781397 18050451

[20] Huang K-M, Cosman P, Schafer WR. Automated detection and analysis of foraging behavior in Caenorhabditis elegans. Journal of Neuroscience Methods. 2008;171(1):153–64. https://doi.org/10.1016/j.jneumeth.2008.01.027. doi: 10.1016/j.jneumeth.2008.01.027 18342950 10.1016/j.jneumeth.2008.01.027

[21] Essmann CL, Elmi M, Shaw M, Anand GM, Pawar VM, Srinivasan MA. In-vivo high resolution AFM topographic imaging of Caenorhabditis elegans reveals previously unreported surface structures of cuticle mutants. Nanomedicine: Nanotechnology, Biology and Medicine. 2017;13(1):183–9. doi: 10.1016/j.nano.2016.09.006 27702605 10.1016/j.nano.2016.09.006

[22] Section II Cuticle. In: Riddle DL, Blumenthal T, Meyer BJ, Priess JR, editors. *C elegans* II. 2nd edition ed: Cold Spring Harbor Laboratory Press; 1997. 21413221

[23] Johnstone IL. The cuticle of the nematode Caenorhabditis elegans: A complex collagen structure. BioEssays. 1994;16(3):171–8. doi: 10.1002/bies.950160307 8166670 10.1002/bies.950160307

[24] Ageing: Lessons from *C*. *elegans*. 1st edition ed. Olsen A, Gill MS, editors: Springer; 2017.

[25] Laissue PP, Alghamdi RA, Tomancak P, Reynaud EG, Shroff H. Assessing phototoxicity in live fluorescence imaging. Nat Meth. 2017;14(7):657–61. doi: 10.1038/nmeth.4344 28661494 10.1038/nmeth.4344

[26] Prevedel R, Yoon Y-G, Hoffmann M, Pak N, Wetzstein G, Kato S, et al Simultaneous whole-animal 3D imaging of neuronal activity using light-field microscopy. Nature Methods. 2014;11(7):727–30. doi: 10.1038/nmeth.2964. WOS:000338321400014. 24836920 10.1038/nmeth.2964 PMC4100252

[27] Shaw M, Elmi M, Pawar V, Srinivasan MA. Investigation of mechanosensation in C. elegans using light field calcium imaging. Biomedical Optics Express. 2016;7(7):2877–87. doi: 10.1364/BOE.7.002877. PMC4948637. 27446713 10.1364/BOE.7.002877 PMC4948637

[28] Wei G, Cosman P, Berry CC, Zhaoyang F, Schafer WR. Automatic tracking, feature extraction and classification of C. elegans phenotypes. IEEE Transactions on Biomedical Engineering. 2004;51(10):1811–20. doi: 10.1109/TBME.2004.831532 15490828 10.1109/TBME.2004.831532

[29] Broxton M, Grosenick L, Yang S, Cohen N, Andalman A, Deisseroth K, et al Wave optics theory and 3-D deconvolution for the light field microscope. Optics Express. 2013;21(21):25418–39. doi: 10.1364/OE.21.025418. WOS:000326085600097. 24150383 10.1364/OE.21.025418 PMC3867103

[30] Zhang M, Geng Z, Pei R, Cao X, Zhang Z. Three-dimensional light field microscope based on a lenslet array. Optics Communications. 2017;403(Supplement C):133–42. https://doi.org/10.1016/j.optcom.2017.07.026.

[31] Cong L, Wang Z, Chai Y, Hang W, Shang C, Yang W, et al Rapid whole brain imaging of neural activity in freely behaving larval zebrafish (Danio rerio). eLife. 2017;6:e28158 doi: 10.7554/eLife.28158. PMC5644961. 28930070 10.7554/eLife.28158 PMC5644961

[32] Broekmans OD, Rodgers JB, Ryu WS, Stephens GJ. Resolving coiled shapes reveals new reorientation behaviors in C. elegans. eLife. 2016;5:e17227 doi: 10.7554/eLife.17227 27644113 10.7554/eLife.17227 PMC5030097

