# Supplementary material for: Three-dimensional behavioural phenotyping of freely moving C. elegans using quantitative light field microscopy
Source: PLoS One. 2018 Jul 11;13(7):e0200108. doi: 10.1371/journal.pone.0200108 (PMC6040744; doi:10.1371/journal.pone.0200108)
Supplement: S1 Text — A discussion of how microscope design parameters affect the lateral spatial resolution, field of view and effective depth of field in a light field microscope. (DOCX) [file pone.0200108.s001.docx]

## Spatial resolution and working volume in LFM

The effective spatial resolution and working volume of a light field microscope are determined by the choice of objective lens and MLA [[1](#_ENREF_1)]. We used a 10x/0.3 water immersion objective lens (UMPLFLN 10XW, Olympus) and an f/20 MLA comprised of square microlenses with a pitch of 125 μm; based on the Nyquist sampling criterion this gives an effective lateral resolution of 25 μm. At f/16.7 the image side f-number of the objective lens is somewhat smaller than the f-number of the microlenses so, to prevent overlapping of the subimages generated by the MLA, the NA of the condenser was reduced to 0.25 using an adjustable iris. The 2048x2048 array of 6.5 μm pixels in the camera image sensor resulted in a field of view of 1.3 mm x 1.3 mm, which is large enough to accommodate an adult wild type worm. With a microlens pitch of 125 μm, each subimage in a raw captured light field has a diameter of *N_u_* = 19.2 camera pixels.

The ability of the system to detect depth differences using the defocus depth cue is related to the depth of field (DOF) of the refocused images. This is significantly larger than the DOF of a corresponding conventional imaging system due to the decreased lateral resolution caused by undersampling of the intermediate image by the microlenses, and is given by [[1](#_ENREF_1)] ${DOF}_{defoc}=\left( 2+N_{u} \right)\lambda n/2{NA}^{2}$. For our system this is equal to 65.0 μm (at *λ* = 0.55 μm). The DOF of the pinhole views is larger still due to the effective reduction in lens aperture and is given by ${DOF}_{ph}=\left( 2+{N_{u}}^{2} \right)\lambda n/2{NA}^{2}$, which is equal to 0.8 mm. This latter DOF sets the axial range over which digital refocusing is possible without a decrease in image quality (lateral spatial resolution). In practice (see Fig. 3 in the manuscript) we found that we could detect depth differences over a somewhat larger range.

## References

1. Levoy M, Ng R, Adams A, Footer M, Horowitz M. Light field microscopy. Acm Transactions on Graphics. 2006;25(3):924-34. doi: 10.1145/1141911.1141976. PubMed PMID: WOS:000239817400054.
