# Supplementary figures and images for: Three-dimensional behavioural phenotyping of freely moving C. elegans using quantitative light field microscopy
Source: PLoS One. 2018 Jul 11;13(7):e0200108. doi: 10.1371/journal.pone.0200108 (PMC6040744; doi:10.1371/journal.pone.0200108)

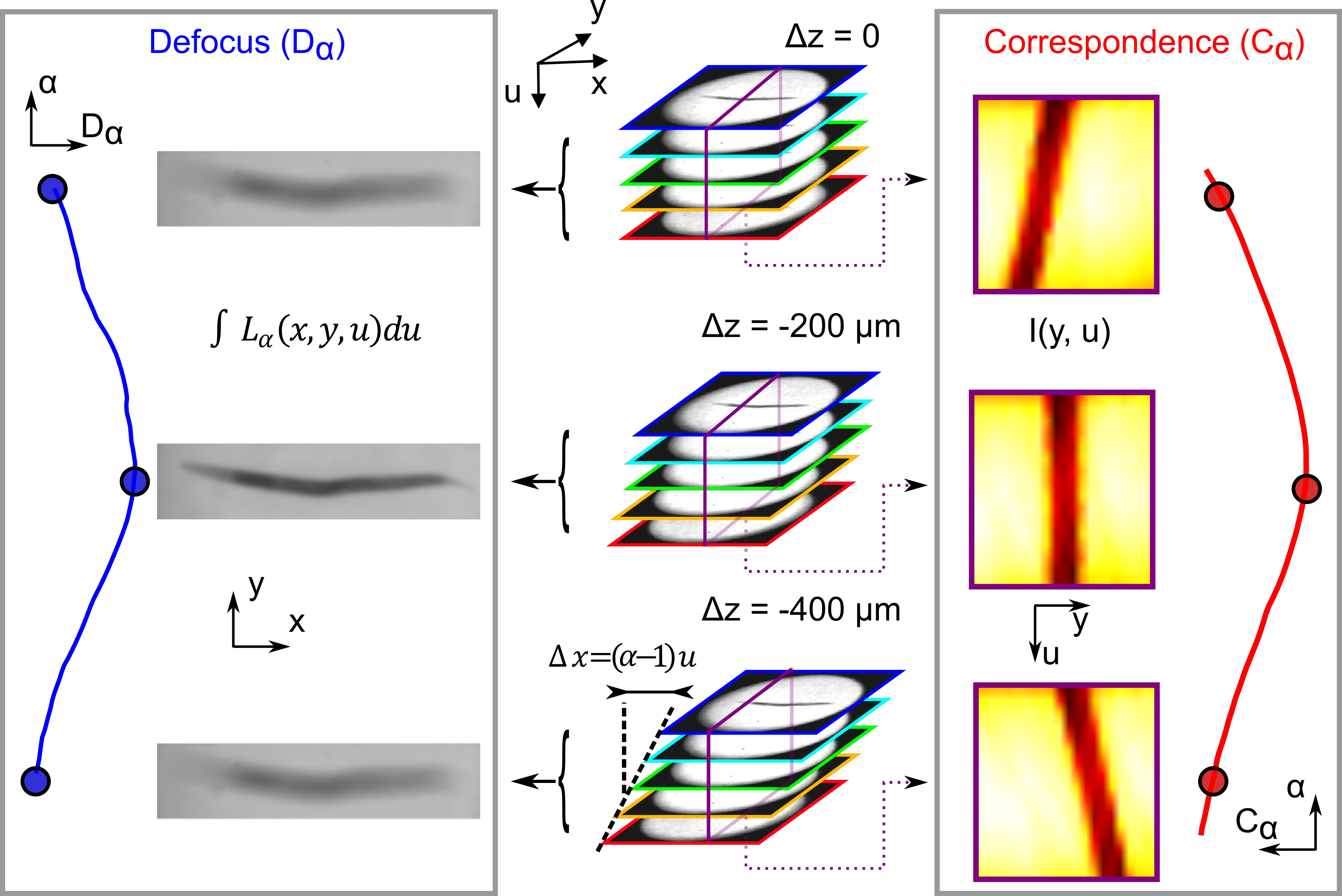

Supplement: S1 Fig — The central part of the figure depicts the processing of light field shearing in 3D (equivalent to laterally shifting each pinhole view). The left panel shows the refocused images created by integrating the sheared light field over all view directions (u, v). The right panel shows three 2D epipolar images, with each row of pixels corresponding to a line profile through a single pinhole image. When the light field is sheared by the amount corresponding to the depth of the object, its position does not change with view angle and it appears as a vertical line in the epipolar image. In this example defocus and correspondence responses (blue and red curves), both indicate the worm body is offset by ~-200 μm from the native object plane. (TIF) [file pone.0200108.s002.tif]
